# Site-specific conjugation of antifreeze proteins onto polymer-stabilized nanoparticles†
†Electronic supplementary information (ESI) available: Synthetic and analytical details. See DOI: 10.1039/c8py01719k


**DOI:** 10.1039/c8py01719k

**Published:** 2019-01-31

**Authors:** Laura E. Wilkins, Muhammad Hasan, Alice E. R. Fayter, Caroline Biggs, Marc Walker, Matthew I. Gibson

**Affiliations:** a Department of Chemistry , University of Warwick , Coventry , CV4 7AL , UK . Email: m.i.gibson@warwick.ac.uk; b Department of Physics , University of Warwick , Coventry , CV4 7AL , UK; c Warwick Medical School , University of Warwick , Coventry , CV4 7AL , UK

## Abstract

Antifreeze proteins (AFPs) have many potential applications, ranging from cryobiology to aerospace, if they can be incorporated into materials. Here, a range of engineered AFP mutants were prepared and site-specifically conjugated onto RAFT polymer-stabilized gold nanoparticles to generate new hybrid multivalent ice growth inhibitors. Only the SNAP-tagged AFPs lead to potent ‘antifreeze’ active nanomaterials with His-Tag capture resulting in no activity, showing the mode of conjugation is essential. This versatile strategy will enable the development of multivalent AFPs for translational and fundamental studies.

## Abstract 2

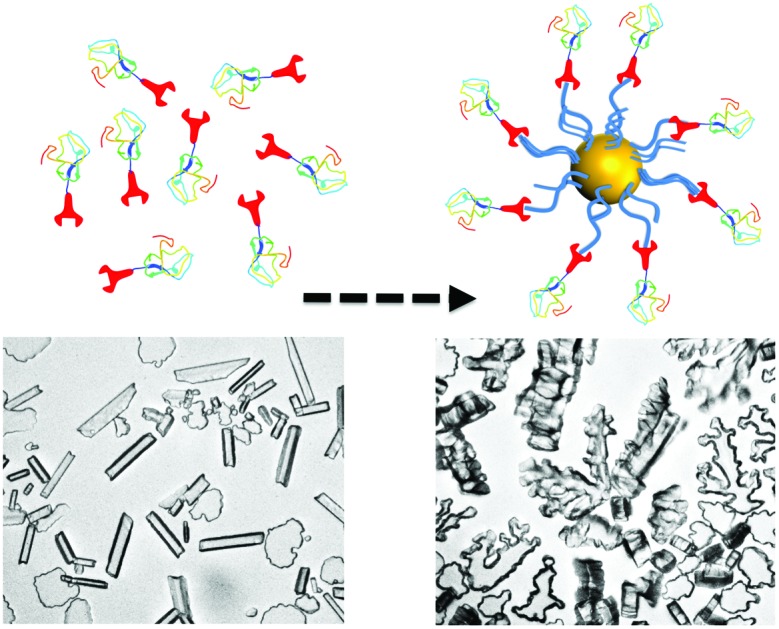
Antifreeze proteins are site-specifically conjugated onto polymer-stabilised gold nanoparticles, resulting in hybrid materials capable of modulating ice growth processes.

## 

Antifreeze (glyco)proteins (AF(G)Ps), are found in a diverse range of organisms^1^ including polar fish,^2^ insects^3^ and plants,^4^ as well as ice-binding polysaccharides in insects.^5^ There is significant interest in the use of these proteins for low temperature applications from aeronautical engineering to wind turbines and for the cryopreservation of donor cells and tissues.^6^,^7^ For this to be a reality, the AFPs need scalable syntheses (or mimics^8^,^9^) and methods to incorporate them into more complex devices or coatings.^10^,^11^ AFPs have three main effects, of ice recrystallisation (growth) inhibition (IRI), non-colligative depression of the freezing point leading to a thermal hysteresis (TH) gap and dynamic ice shaping (DIS). To enable interaction with the dynamic surface of ice, most AFPs (but not the more flexible antifreeze glycoproteins^12^) have a defined ice-binding face^1^ which can anchor them directly or *via* ordered clathrate water.^13^–^16^ A distinct class of AFPs are the hyperactive AFPs.^17^,^18^ The increased TH activity of hyperactive AFPs is linked to their binding of both prism and basal planes of ice, compared to just the prism plane for standard AFPs.^19^,^20^ Increasing the concentration of particular type I AFPs has been observed to lead to oligomerization to a tetramer and the onset of hyperactivity, linked to its supramolecular assembly.^21^ Davies and coworkers assembled 6–11-mers of AFP type III on PAMAM dendrimers to mimic this oligomerization. On a per-protein basis there was only a small increase in IRI, but the ability to span multiple ice faces increased the TH activity.^22^ Synthetic mimics of AFPs also show strong molecular weight dependence on activity, with longer polymers (such as poly(vinyl alcohol) having significantly higher IRI activity^23^–^25^ as do supramolecular safranine-O based mimics.^26^

Despite the evidence for increasing the valency of AFPs to modulate activity there remain few reports of multivalent display, in part due to the challenges of site-specific protein conjugation.^10^,^27^,^28^ Traditional approaches to combine polymers with proteins involve targeting unpaired cystine residues.^29^,^30^ Unnatural amino acids for bioconjugation can be incorporated in a site-specific fashion by the AMBER stop codon.^31^ Johnsson and co-workers have developed recombinantly expressible ‘SNAP-tags’ based on O^6^alkylguanine-DNA alkyltransferase.^32^ By attaching the tag as a fusion protein a covalent bond can be formed to any surface bearing benzylguanine without any unnatural amino acids. The commonly used hexa-histidine purification tag can also facilitate ionic-conjugation.^33^

Here AFPs are conjugated, by site-specific methods, onto nanoparticles to generate hybrid ice growth inhibiting materials to mimic the multivalent presentation of hyperactive antifreeze proteins and aid the application of these exciting proteins (Fig. 1).

**Fig. 1:**
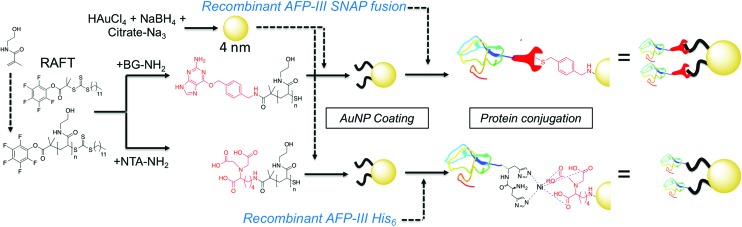
Synthesis of gold/polymer/antifreeze protein hybrid particles. NTA = Nitrilotriacetic acid; BG = O^6^-benzylguanine; His_6_ = hexa-histidine tag. Red ‘wrench’ represents the SNAP-tag.

RAFT (reversible addition fragmentation chain transfer) polymerization was employed to synthesize telechelic poly(hydroxyethyl acrylamide), pHEA, bearing a pentafluorophenyl (PFP) ester at the α-terminus and a trithiocarbonate at the ω-terminus.^34^ These polymers were characterized by SEC, ^1^H, ^19^F NMR and IR (Table 1). The PFP group was substituted by addition of amino-benzylguanine (BG) (for SNAP conjugation^32^) or tris-NTA amine (for His-Tag capture). Successful conjugation was confirmed by ^19^F NMR (Fig. 2B) as well as by IR. By using an excess of the amine, the RAFT agent end group was displaced to reveal a thiol for gold particle conjugation. It was also attempted to introduce a maleimide onto the particles, but this did not lead to stable particles (see ESI† for details) so was not taken further.

**Fig. 2:**
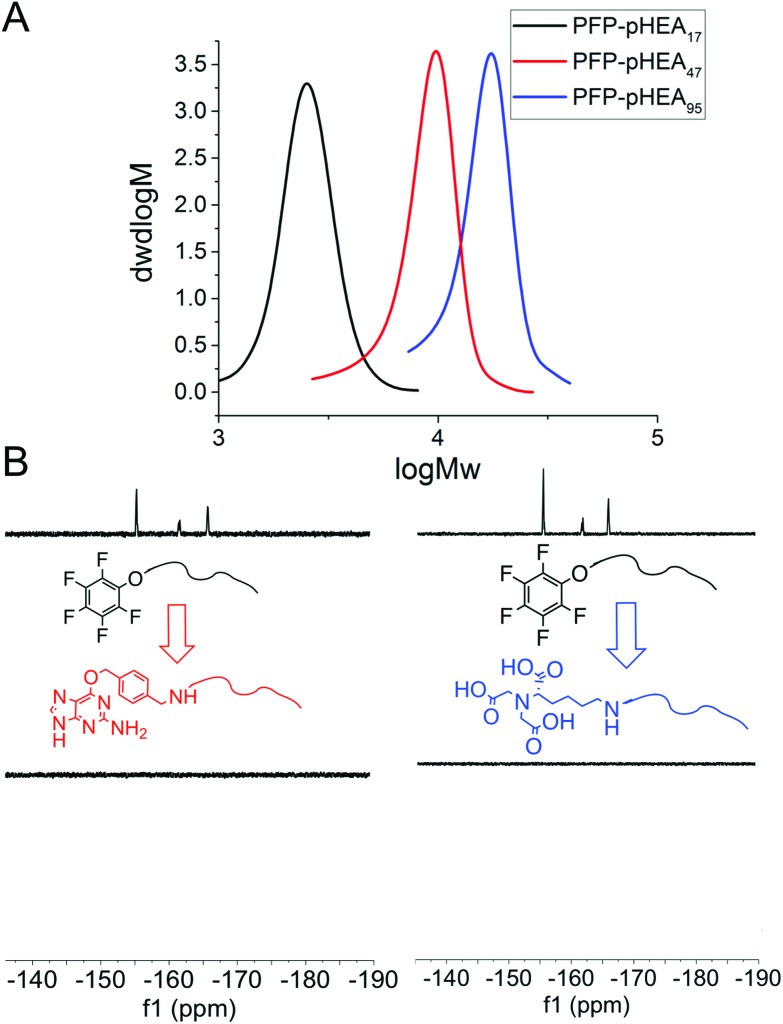
(A) SEC analysis of PFP-pHEAs; (B) ^19^F-NMR analysis of end group displacement.

**Table 1:** Precursor polymers synthesized

Polymer	[M]/[CTA] (–)	Conv.^*a*^ (%)	DP_*n*_ (–)	*M*_n(THEO)_ ^*a*^ (g mol^–1^)	*M*_n(SEC)_ ^*b*^ (g mol^–1^)	*Đ* ^*b*^ (–)
PFP-pHEA_95_	103	93	95	11 000	15 000	1.3
PFP-pHEA_45_	47	94	45	5700	8300	1.1
PFP-pHEA_17_	26	64	17	2500	3000	1.1

^*a*^Determined by ^1^H NMR.

^*b*^Determined by SEC against PMMA standards.

4 nm citrate-stabilized gold nanoparticles (AuNP) were synthesized by NaBH_4_ reduction of HAuCl_4_ and subsequently functionalized with thiol-terminated polymers by mixing, followed by centrifugal dialysis.^35^ The nanoparticles were characterized by transmission electron microscopy (Fig. 3A–C), dynamic light scattering (Fig. 3E) and UV-Vis confirming successful addition of the polymer. X-Ray photo electron spectroscopy (XPS) showed a clear increase in the nitrogen N 1s signal following coating (Fig. 3D). Screening experiments showed that NTA-pHEA gave more stable particles when *M*_n_ = 3000 g mol^–1^ and for BG-pHEA = 10 000 g mol^–1^ and hence these chain lengths were used from this point onwards.

**Fig. 3:**
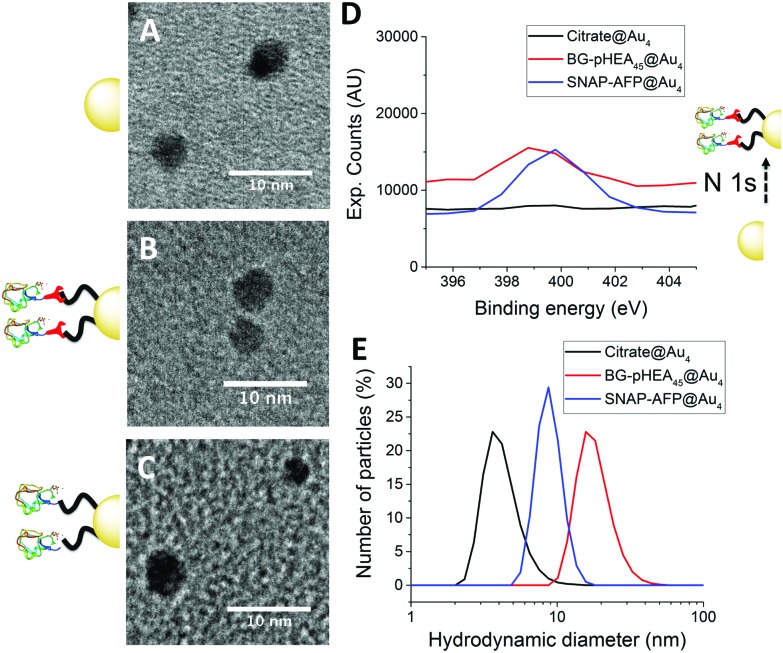
Nanoparticle characterization. (A–C) Transmission electron micrographs of citrate@Au_4_, SNAP-AFP-BG-pHEA_45_@Au_4_ and His-AFP-Ni-NTA-pHEA_17_@Au_4_ respectively; (D) N 1s peak from XPS upon conjugation of SNAP-AFP; (E) diameter from dynamic light scattering.

Recombinant AFP type III (from ocean pout) were produced by expression in *Escherichia coli*, and purified by nickel affinity chromatography and HPLC. Three mutants were produced bearing N-terminal modifications of hexa-histidine (His-AFP), SNAP (SNAP-AFP) and cysteine (Cys-AFP). Direct conjugation of the SNAP-AFP was achieved by incubation with the BG-pHEA_45_@AuNP_4_ in buffer. NTA-pHEA_17_@AuNP_4_ was first activated by addition of NiCl_2_ followed by His-AFP. Direct Cys-AFP immobilization onto AuNPs was unsuccessful (see ESI†). Particles were further purified by centrifugal dialyses and removal of non-conjugated residual protein confirmed by nanodrop absorption analysis on residual washings. Following conjugation the zeta-potential became less negative and XPS confirmed a further increase in the nitrogen concentration upon protein conjugation (Table 2).

**Table 2:** Nanoparticle characterization

Particle	*D*_h_ ^*a*^ (nm)	*D*_TEM_ ^*b*^ (nm)	*ζ* potential (mV)
Citrate@Au_4_	4.3	3.9 ± 0.7	–19.4 ± 2.7
NTA-pHEA_17_@Au_4_	8.5	3.7 ± 0.8	–15.9 ± 4.8
Ni-NTA-pHEA_17_@Au_4_	7.7	3.9 ± 0.8	–6.16 ± 1.1
His-AFP-Ni-NTA-pHEA_17_@Au_4_	8.9	4.3 ± 0.9	–7.03 ± 1.3
BG-pHEA_45_@Au_4_	16.5	3.9 ± 1.0	–10.2 ± 1.9
SNAP-AFP-BG-pHEA_45_@Au_4_	8.92	4.0 ± 1.1	–4.35 ± 0.9

^*a*^Hydrodynamic diameter from DLS.

^*b*^Gold core diameter, average of >100 particles by TEM.

With these site-specifically attached AFP/nanoparticle conjugates to hand, their IRI activity could be assessed by the ‘splat’ assay. In brief, a polynucleated wafer of ice crystals is seeded from a solution containing the compounds of interest and the ice crystals annealed at–8 °C for 30 minutes. After this time the average ice crystal size is evaluated and compared to a negative control of buffer alone: smaller ice crystal sizes signify more IRI activity. The two AFP mutants were very potent inhibitors, preventing all growth below 0.01 mg mL^–1^. There was no significant difference between the two mutants, showing the fusions were tolerated in terms of retention of function. His-AFP-Ni-NTA-pHEA_17_@Au_4_ was found to be surprisingly inactive as an ice growth inhibitor with no activity up to 1 mg mL^–1^ (AFP concentration). This could be due to the dynamic nature of the His-NTA interaction or incorrect orientation of the AFP on the surface. This also shows that designing a multivalent AFP is non-trivial, with the correct chemistry being essential. Previous studies suggest excess charge does not affect AFP activity and was ruled out as the cause.^36^ In contrast, the covalent hybrid nanoparticle SNAP-AFP-BG-pHEA_45_@Au_4_ was found to be a very potent IRI, Fig. 4B. In terms of total AFP concentration (to enable multivalent effects to be seen) the nanoparticles essentially identical activity compared to SNAP-AFP alone. If considered on a molar basis (as has been done by Davies *et al.* for dendrimer AFPs^22^) then the particles are more active. AFPs are known to become hyperactive when they oligomerize, but such enhancement was not seen here due to the different 3-D placement of the AFPs.^21^ Using a nanoliter osmometer the thermal hysteresis (TH) gap (non-colligative freezing point depression) was determined. TH is closely linked to the ability to bind specific ice faces (in this case the prism plane) hence providing additional information about the functionality of the particles; the TH is unique to antifreeze proteins, and is defined as the difference between the freezing and melting points. It was observed that the AFP and SNAP-AFP nanoparticles have very similar TH gaps, of 1.4 °C at 7.6 mg mL^–1^. As with the IRI measurements, this confirms that all activity is retained despite the major structural modifications and that the placement of the AFPs *via*-polymeric linkers is tolerated. As the effective AFP concentration in solution when immobilized on NPs is lower, this suggests that the particles can bind multiple planes of ice simultaneously. To probe this dynamic ice shaping experiments were conducted in sucrose solution (as described previously^28^). Compared to SNAP-AFP alone (Fig. 4H), the SNAP–AuNP conjugates resulted in more faceting of the ice crystals, consistent with them being able to bind more ice faces simultaneously.^22^

**Fig. 4:**
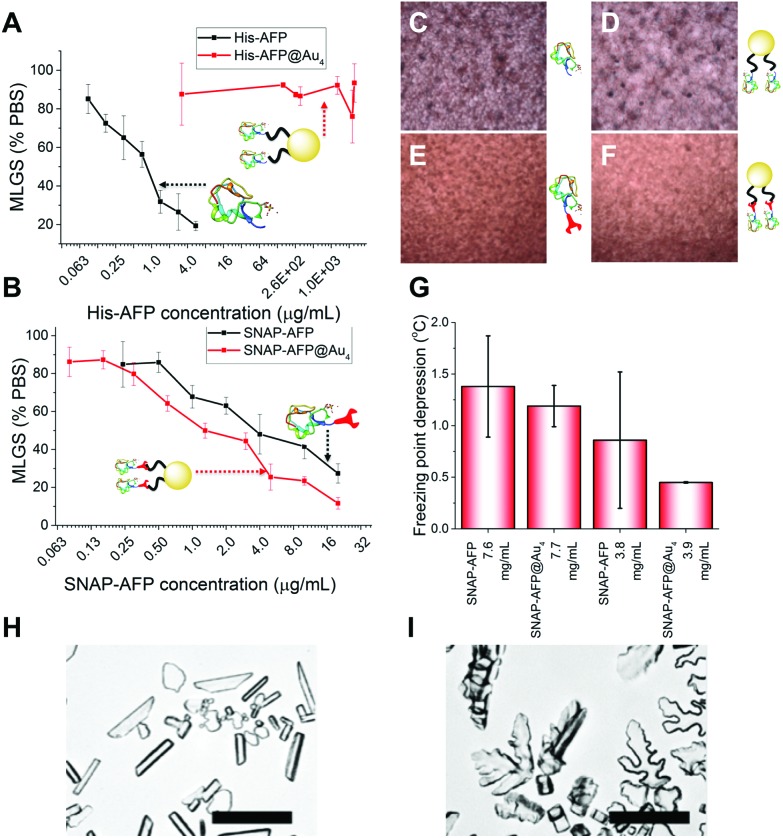
(A, C, D) IRI activity of His-AFP and conjugates by MLGS (mean length grain size); (B, E, F) IRI activity of SNAP-AFP and conjugates; (G) thermal hysteresis showing freezing point depression in SNAP-AFP and conjugate samples; ice crystal morphology of (H) SNAP-AFP; (I) SNAP-AFP-BG-pHEA_45_@Au_4_. Scale bar = 100 μm.

## Conclusions

In conclusion, we report the synthesis of hybrid nanoparticle–AFP conjugates, making use of polymer-stabilized gold nanoparticles and protein engineering to enable precision-conjugation. Hetero-telechelic polymers were obtained by RAFT, enabling a nanoparticle immobilization moiety (thiol) and distal protein capture (nickel NTA or O^6^-benzyl-guanine) moiety to be installed. The polymer coating is essential to enable conjugation in buffer, avoiding nanoparticle aggregation. It was found that the SNAP-tag (covalent) approach lead to the most active particles compared to oligohistidine capture. The observed ice recrystallisation inhibition and thermal hysteresis activity was comparable to free antifreeze proteins on a valency-corrected basis confirming retention of activity in this format. Other conjugation methods based on ionic interactions did not give stable particles or resulted in loss of activity. These results demonstrate that antifreeze protein can be incorporated into more complex assemblies to facilitate their application in colloid science or for fundamental studies exploiting nanoparticle cores for bioimaging and tracking.

## Data access statement

The research data supporting this publication can be found at http://wrap.warwick.ac.uk.

## Conflicts of interest

There are no conflicts to declare.

## Supplementary Material

Supplementary information
