# Supplementary material for: Site-specific conjugation of antifreeze proteins onto polymer-stabilized nanoparticles
Source: Polym Chem. 2019 Jan 31;10(23):2986–90. doi: 10.1039/c8py01719k (PMC6592154; doi:10.1039/c8py01719k)
Supplement: Supplementary file 1 [file PY-010-C8PY01719K-s001.pdf]

Electronic Supplementary Material (ESI) for Polymer Chemistry.
This journal is © The Royal Society of Chemistry 2019

Supporting information for:

Site-Specific Conjugation of Antifreeze Proteins onto Polymer-Stabilized Nanoparticles

Laura E. Wilkins,[a] Muhammad Hasan,[b] Alice E.R. Fayter,[a], Caroline I. Biggs,[a] Marc Walker,[c] Matthew I. Gibson*,[a,b]

[a] Department of Chemistry, University of Warwick, Coventry, CV4 7AL, United Kingdom

[b] Warwick Medical School, University of Warwick, Coventry, CV4 7AL, United Kingdom

[c] Department of Physics, University of Warwick, Coventry, CV4 7AL, United Kingdom

CORRESPONDING AUTHOR DETAILS

E-mail: m.i.gibson@warwick.ac.uk

Materials

Ultra-pure water with resistance < 18 Ω, was obtained from a Milli-Q© Integral Water Purification System. All chemicals were purchased from Sigma-Aldrich or Thermo Fischer Scientific and used as supplied unless otherwise stated. 6-((4-(aminomethyl)benzyl)oxy)-7H-purin-2-amine was purchased from Carbosynth. THF, DMF and ethyl acetate were purchased from Fisher. For washing of AuNPs, Amicon Ultra-0.5 centrifugal filter units with Ultracel-30 membrane were used. 40 nm citrate-stabilised gold colloid solution was purchased from BBI solutions. The pET20b-AFPIII plasmid encoding for a hexahistidine-tagged AFPIII from ocean pout (*rQAE isoform*, M1.1HISPET20b) was kindly provided by Peter Davies (Queens University, Kingston, Canada). Competent *Escherichia coli* BL21(DE3) cells were sourced from New England Biolabs. IMAC Sepharise 6 Fast Flow columns, HiLoad 16/600 Superdex 75 pg gel filtration columns and PD10 desalting columns were purchased from GE Healthcare and used according to manufacturer's instructions. Thermo Scientific Pierce BCA assay kit was used according to the manufacturer's protocols to determine all protein concentrations. Phusion® High-Fidelity DNA Polymerase and pSNAP-tag® (T7)-2 vector were purchased from New England Biolabs.

Analytical Methods

^{1}H, ^{13}C and ^{19}F-NMR spectra were obtained using a Bruker DPX-400 NMR Spectrometer; all chemical shifts are reported in ppm (δ) relative to residual non-deuterated solvent. Mass spectrometry was carried out in pure methanol or water on the Agilent 6130B ESI-Quad instrument using electrospray in positive mode. FTIR spectroscopy was carried out on a Bruker Vector 22 FTIR spectrometer with a Golden gate diamond attenuated total reflection cell. SEC (GPC) measurements were carried out on an Agilent 390-LC MDS instrument equipped with a dual angle light scatter

(LS), 2 x PLgel Mixed D columns (300 x 7.5 mm) and a PLgel 5 µm guard column. The eluent was DMF with 5 mmol NH_4BF_4 additive. Samples were run at 1ml/min at 50. Poly(methyl methacrylate) standards (Agilent EasyVials) were used for calibration between 955,000 – 550 $gmol^{-1}$. Analyte samples were filtered through a nylon membrane with 0.22 μm pore size before injection. Molar mass ($Mn_{,SEC}$) and dispersity (*Đ*) values of synthesized polymers were determined by conventional calibration against PMMA standards using Agilent GPC/SEC software. Nanoparticle size was determined using Dynamic Light Scattering (DLS), performed on a Malvern Instruments Zetasizer Nano- ZS with 4mW HeNe laser 632.8 nm. UV/Vis spectroscopy, optical density and fluorescence plate readings were performed on a BioTek Synergy HT Microplate Reader. UV-vis spectroscopy was used to determine nanoparticle size according to a method developed by Haiss et al.[1] Transmission electron microscopy (TEM) was performed on a JEOL 2100 LaB6 high-resolution microscope. X-ray photoelectron spectroscopy (XPS) was carried out on the Kratos Axis Ultra with a delay-line detector. Bacterial cell lysis was performed using a STANSTED 'Pressure Cell' FPG12800 homogeniser. WAXS experiments were performed using a Xenocs Xeuss 2.0 equipped with a micro-focus Cu K_α source collimated with Scatterless slits. The scattering was measured using a Pilatus 100k detector with a pixel size of 0.172 µm x 0.172 µm. The distance between the detector and the sample was calibrated using silver behenate ($AgC_{22}H_{43}O_2$), giving a value of 0.161(3) m. The detector was fixed at an angle of 36° giving a 2θ range of 18.5 to 47.5°, WAXS experiments used 1.0 mm borosilicate capillaries (Capillary Tube Supplies Ltd) and recorded patterns from 10 to 45 ° at temperatures between -35 and 10 °C. Thermal hysteresis measurements were performed using a Clifton nanolitre osmometer by Prof Peter Davies, Queens University Canada and using standard procedures [2].

Synthetic and Experimental Procedures

Synthesis of 4 nm gold nanoparticles

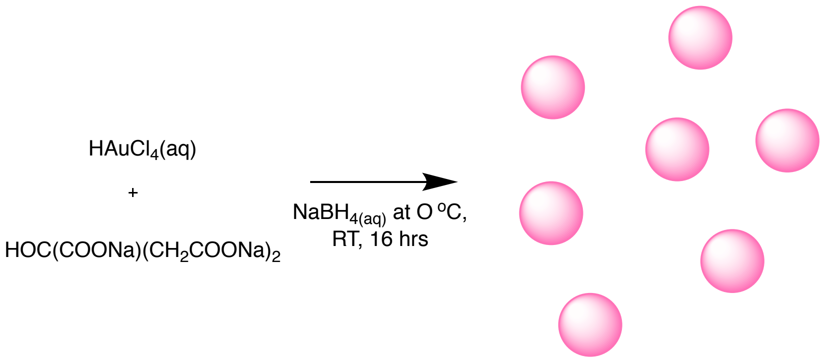

AuNPs were synthesized based on a method by Ieong et al.[3] All glassware was washed with *aqua regia* **[CAUTION. Extreme care must be taken when handling this reagent]**[4] prior to commencing the experiment, to reduce unwanted nucleation from impurities on the surface. 240 mL of a 0.21 mmol.L^{-1}, (0.08 mg.mL^{-1}) aqueous solution of $HAuCl_4$ was prepared at room temperature. To this was added 13.8 mg (0.05 mmol) of trisodium citrate to give a final citrate concentration of 0.21 mmol.L^{-1}. 5 mL of an ice-cold 0.1 M (0.5 mmol, 18.5 mg) solution of $NaBH_4$ was added to the gold/citrate solution and stirred at room temperature overnight. Assuming complete reduction of the $HAuCl_4$ to gold particles, the total gold concentration in the final solution was 0.21 mmol.L^{-1} (0.04 mg/mL). As these small AuNPs did not pellet upon centrifugation, small AuNPs were isolated by taking the supernatant after centrifugation at 13.2 k RPM for 10 mins at 25 °C. Nanoparticle size and dispersity was measured by TEM, UV-Vis spectroscopy and size and zeta potential measurements by DLS.

Nanoparticle diameter (UV-vis): 3-4 nm; TEM: 3.9 nm (standard deviation 0.74 nm).

Synthesis of 2-(dodecylthiocarbonothioylthio)-2-methylpropionic acid (DMP/DDMAT)

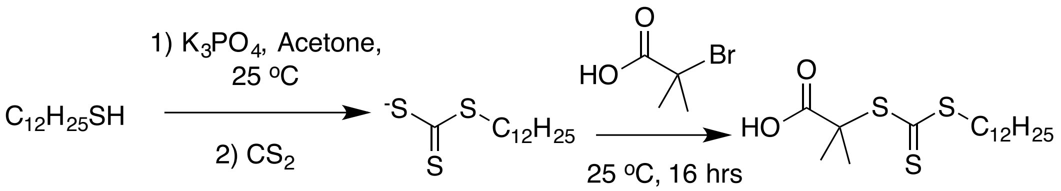

Dodecane thiol (4.75 mL, 19.8 mmol) was added dropwise to a stirred suspension of K_3PO_4 (4.02g, 18.9 mmol) in acetone (60 mL). The reaction vessel was placed in an ice bath. Carbon disulfide (3.20 mL, 53.0 mmol) was added and the solution turned bright yellow, but was still cloudy. After stirring for ten minutes, 2-bromo-2-methylpropionic acid (3.00 g, 18.0 mmol) was added and a precipitation of KBr was noted. The ice bath was removed after 10 minutes and the reaction was left stirring at room temperature for 16 hours. Solvent was removed *in vacuo* and the residue was extracted into DCM (2 x 50 mL) from 1 M HCl (100 mL). The organic extracts were further washed with water (100 mL) and brine (100 mL) and dried over $MgSO_4$. Recrystallisation from n-hexane yielded a bright yellow solid (1.80 g, 27.5%).

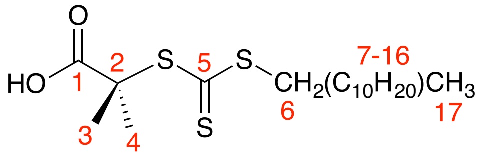

^{1}H NMR (400 MHz, CDCl3) δ_{ppm}: 3.28 (2H, t, J_{HH}=7.5, H6); 1.66 (6H, s, H3/4); 1.10-1.25 (20H, alkyl, H7-16); 0.79 (3H, m, H17).

^{13}C NMR (400 MHz, CDCl3) δ_{ppm}: 221 (C5); 178 (C1); 55.5 (C2); 37 (C7); 32 (C6); 30, 29.5, 29.5, 29.4, 29.1, 29.0, 27.8 (C8-15); 25.2 (C3/4); 22.7 (C16); 14.1 (C17).

FTIR (solid, ν_{max}/cm^{-1}): 2910 (CH_2); 1710 (C=O); 1440 (C-C); 1305 (C-O); 1070 (S-(C=S)-S).

ESI-MS, positive mode (m/z): 365.2 ($M+H^+$, expected 365.63), 387.1 ($M+Na^+$, expected 387.61).

Synthesis of pentafluorophenyl 2-(dodecylthiocarbonothioylthio)-2-methylpropionic acid (PFP-DMP/PFP-DDMAT)

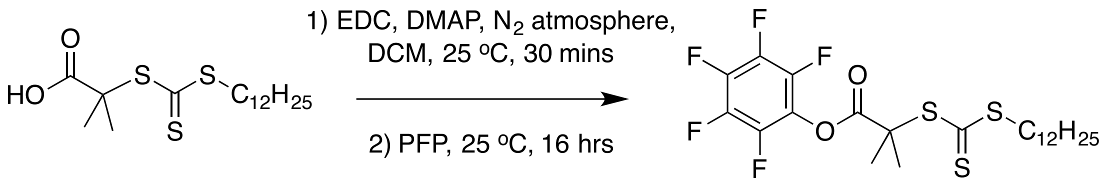

2-(Dodecylthiocarbonothioylthio)-2-methylpropionic acid (DMP) (0.500 g, 1.37 mmol), *N*-(3-dimethylaminopropyl)-*N'*-ethylcarbodiimide hydrochloride (EDC) (0.390 g, 2.05 mmol), and 4-(dimethylamino)pyridine (DMAP) (0.250 g, 2.05 mmol) were dissolved in DCM (50 mL) and stirred for 20 minutes under N_2. Pentafluorophenol (PFP) (0.780 g, 4.24 mmol) in 5 mL DCM was added. The reaction was stirred overnight at room temperature. The reaction was washed with 3 M HCl (100 mL), 1 M $NaHCO_3$ (100 mL) and brine (100 mL), dried over $MgSO_4$, filtered and then concentrated *in vacuo* to evolve a yellow solid with melting point close to room temperature (0.437 g, 60.2%).

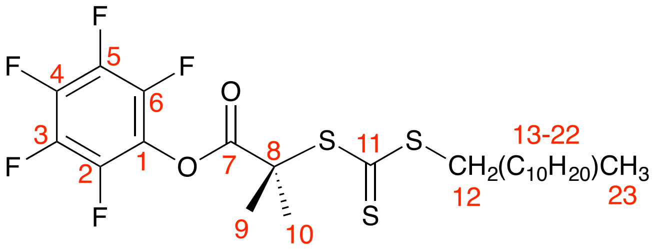

^1H NMR (300 MHz, CDCl3) δ_{ppm}: 3.24 (2H, t, J_{HH}=7.4 x 2, H12); 1.62 (6H, m, H9/10); 1.26 (20H, alkyl, H13-22); 0.81 (3H, m, H23).

^{19}F NMR (300 MHz, CDCl3) δ_{ppm}: 151.54 (m, F2/6), 157.74 (m, F3/5), 162.3 (m, F4).

FTIR (solid, ν_{max}/cm^{-1}): 2934 (CH_2); 1705 ($C_6F_5C{=}O$); 1439 (C-C); 1260 (C-O); 1080 (S-(C=S)-S).

General Procedure for Synthesis of PFP-terminated poly(hydroxyethyle acrylamide)

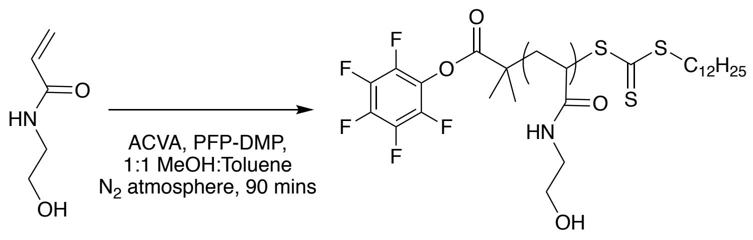

The following procedure describes a reaction with a theoretical degree of polymerization (DP) of 50 repeat units. 4,4-azobis(4-cyanovaleric acid) (5 mg, 0.018 mmol), pentafluorophenyl 2-(dodecylthiocarbonothioylthio)-2-methylpropionic acid (CTA) (47 mg, 0.088 mmol, for the polymer without a PFP-end group, DMP was used instead of PFP-DMP) and *N*-hydroxyethyl acrylamide (1 g, 8.8 mmol) were dissolved in 1:1 methanol: toluene (4 mL) in a glass vial with a stirrer bar. Mesitylene (200 μL) was added and a sample was removed for ^{1}H-NMR analysis in $CDCl_3$. The reaction mixture was degassed by N_2 for 30 minutes, sealed and placed in a 70°C oil bath. After 90 minutes, the solution was opened to air and quenched in $N_{2(l)}$. The polymer (pHEA) was precipitated three times from methanol into diethyl ether to give a light yellow solid.

Conversion (NMR): 94.0%; Mn (theoretical): 5664 g.mol^{-1}; Mn (SEC) 8253 g.mol^{-1}; Mw (SEC) 9231 g.mol-1; Mw/Mn (SEC): 1.12.

^{1}H NMR (300 MHz, CDCl3) δ_{ppm}: 8-8.15 (br s, N-H (H4)); 3.4-3.8 and 3.05-3.2 (2 x m, H5); 1.9-2.35 and 1.4-1.8 (2 x m, H1/2).

^{19}F NMR (300 MHz, CDCl3) δ_{ppm}: 155.29, 161.61, 165.67.

FTIR (solid, ν_{max}/cm^{-1}) = 3300 (N-H and O-H stretch); 2854 (alkyl C-H stretch); 1641 (amide C=O stretch); 1555 (N-H bend); 1443 (alkane); 1225 (C-O stretch); 1060 (C-O stretch); 950 (C-F peak on shoulder of 1060 peak).

Data are shown for the DP-46 polymer.

General Procedure for Synthesis of Amine-modified (BG- or NTA-amine) pHEA polymer

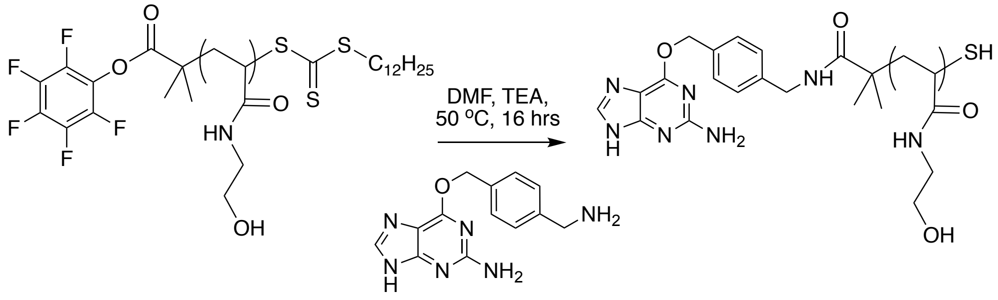

Synthesis of BG-pHEA polymer.

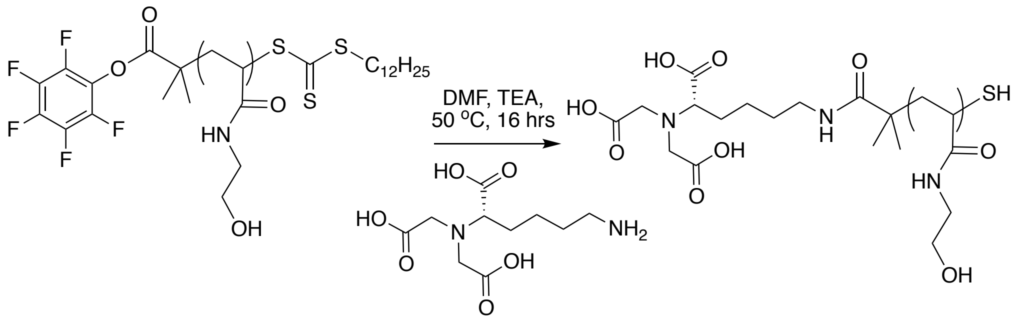

Synthesis of NTA-pHEA polymer.

PFP-pHEA (90.8 mg, 0.018 mmol) and primary amine (6-((4-(aminomethyl)benzyl)oxy)-7H-purin-2-amine (BG) or Nα,Nα-Bis(carboxymethyl)-L-lysine hydrate(NTA)) (0.088 mmol) were dissolved in DMF (5 mL). To the stirred mixture, triethylamine (35 μL) was added, and the reaction was moved to a 50 oil bath

for 16 hours. The polymer was precipitated three times from methanol into diethyl ether to give a sandy yellow solid.

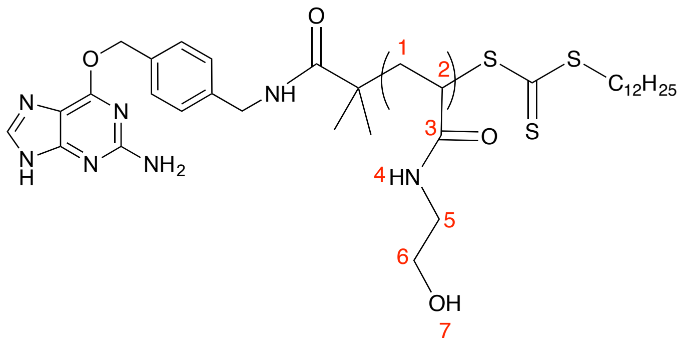

BG-pHEA45 polymer

M_n (SEC) 12960 $g.mol^{-1}$; M_w (SEC) 15977 $g.mol^{-1}$; M_w/M_n (SEC): 1.23.

^{1}H NMR (300 MHz, D_4-MeOH) δ_{ppm}: 4.8-5 (br s, H6), 3.44-3.85 and 3.05-3.22 (2 x br s, H5), 1.94-2.35 and 1.31-1.86 (2 x br s, H1/2).

^{19}F NMR (300 MHz, $CDCl_3$) δ_{ppm}: no peaks visible.

FTIR (solid, ν_{max}/cm^{-1}) = 3300 (N-H and O-H stretch); 2854 (alkyl C-H stretch); 1641 (amide C=O stretch); 1555 (N-H bend); 1443 (C-H alkane); 1225 (C-O stretch); 1060 (C-O stretch) (C-F shoulder on 1060 peak is no longer present).

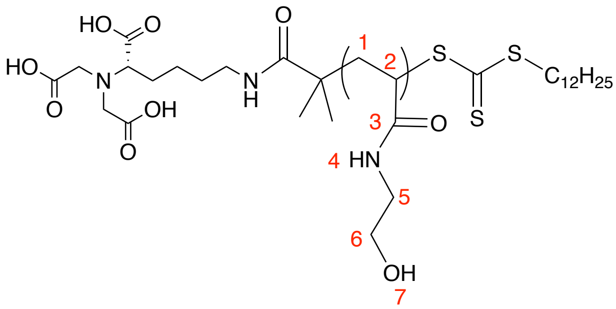

NTA-pHEA17 polymer

M_n (SEC) 3040 $g.mol^{-1}$; M_w (SEC) 3355 $g.mol^{-1}$; M_w/M_n (SEC): 1.10.

^{1}H NMR (300 MHz, D_4-MeOH) δ_{ppm}: 4.8-5 (br s, H6), 3.44-3.85 and 3.05-3.22 (2 x br s, H5), 1.94-2.35 and 1.31-1.86 (2 x br s, H1/2).

^{19}F NMR (300 MHz, $CDCl_3$) δ_{ppm}: no peaks visible.

FTIR (solid, ν_{max}/cm^{-1}) = 3300 (N-H and O-H stretch); 2944 (alkyl C-H stretch); 1627 (amide C=O stretch); 1562 (N-H bend); 1390 (C-H alkane); 1060 (C-O stretch) (C-F shoulder on 1060 peak is no longer present).

General Procedure for Synthesis of Polymer-Coated AuNP (NTA-pHEA$_{17}$-, PFP-pHEA$_{47}$- or BG-pHEA$_{45}$@Au$_4$)

1 mg of polymer (either PFP-pHEA48, BG-pHEA45 or NTA-pHEA6) was added to 1 mL of citrate-Au$_4$ in a 1.5 mL Eppendorf tube. This was left, agitating, for 60 minutes. The solutions were concentrated by centrifugation in Amicon Ultra-0.5 centrifugal filter units with an Ultracel-30 membrane, before being re-dispersed in the same volume of water. The nanoparticles were washed in this manner a further 3 times before being re-dispersed in the same final volume of distilled water and stored in the fridge until required. Nanoparticle size and dispersity was measured by TEM, UV-Vis spectroscopy and size and zeta potential measurements by DLS.

BG-pHEA$_{45}$@Au$_4$: Nanoparticle diameter (UV-vis): 3-4 nm; TEM: 3.87 nm (standard deviation 1.04 nm).

NTA-NTA$_{17}$@Au$_4$: Nanoparticle diameter (UV-vis): 3-4 nm; TEM: 3.70 nm (standard deviation 0.74 nm).

PFP-pHEA$_{47}$-@Au$_4$: Nanoparticle diameter (UV-vis): 3-4 nm; TEM: 4.38 nm (standard deviation 0.68 nm).

Synthesis of Maleimide-pHEA$_{47}$@Au$_4$

NOTE. These particles were found not to be stable in buffer and were not taken forward, but included for completeness.

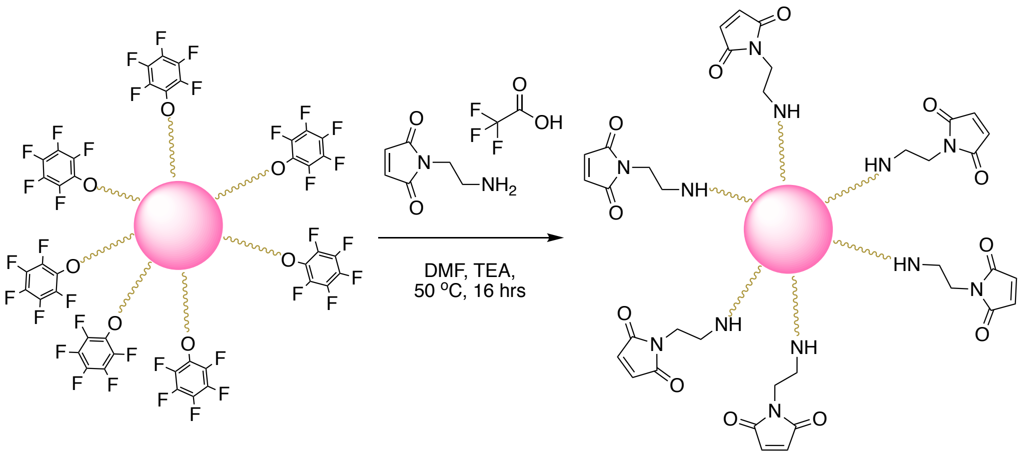

PFP-pHEA$_{47}$@Au$_4$ solution (500 µL) and N-(2-Aminoethyl)maleimide trifluoroacetate salt (0.00088 g, 3.4 µmol) were dissolved in DMF (500 µL) in a 1.5 mL polypropylene microcentrifuge tube and vortexed 30 secs to dissolve. To the stirred mixture, triethylamine (3.5 µL, for 0.5 M solution) was added, and the reaction was moved to a 50 oil bath for 16 hours. The solutions were added to 4.5 mL of H_2O before being concentrated by centrifugation in Amicon Ultra-0.5 centrifugal filter units with an Ultracel-30 membrane. The AuNPs were re-dispersed in 500 µL of PBS buffer, pH 7.5. The nanoparticles were washed by concentration and re-dispersion a further 2 times and stored in the fridge until required. Nanoparticle size and dispersity was measured by TEM, UV-Vis spectroscopy and size and zeta potential measurements by DLS. Nanoparticle diameter (UV-vis): 3-4 nm; TEM: 4.31 nm (standard deviation 0.80 nm). *These quickly aggregated making them non-usable.*

Synthesis of Ni-NTA-pHEA$_{17}$@Au$_4$

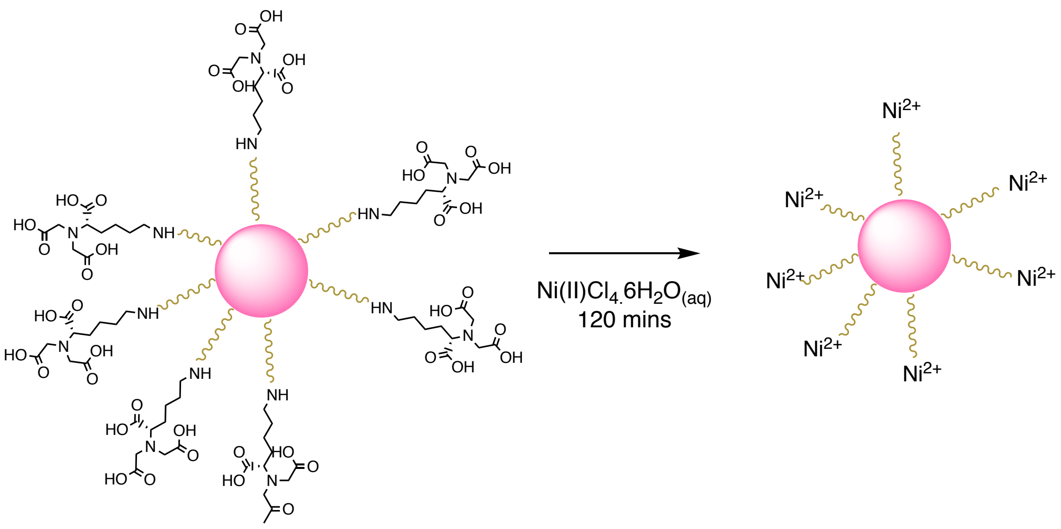

NTA-pHEA$_{17}$@Au$_4$ solution (1 mL) was concentrated by centrifugation in Amicon Ultra-0.5 centrifugal filter units with an Ultracel-30 membrane. To this concentrate was added the same volume Nickel(II) Chloride Hexahydrate (1 mL of 500 µM solution at pH 8 in 10 mM HEPES, 100 mM NaCl buffer). This was left, agitating, for 120 minutes. The solutions were concentrated by centrifugation in Amicon Ultra-0.5 centrifugal filter units with an Ultracel-30 membrane, before being re- dispersed in the same volume of pH 7.5 HEPES/NaCl buffer. The nanoparticles were washed in this manner a further 3 times before being re-dispersed in the same final volume of buffer and stored in the fridge until required. Nanoparticle size and dispersity was measured by TEM, UV-Vis spectroscopy and size and zeta potential measurements by DLS.

Nanoparticle diameter (UV-vis): 3-4 nm; TEM: 3.89 nm (standard deviation 0.75 nm)

General Synthesis of AFP@Au$_4$ (from Ni-NTA-pHEA$_{17}$-, Maleimide-pHEA$_{47}$- or BG-pHEA$_{45}$@Au$_4$)

Ni-NTA-pHEA$_{17}$-, Maleimide-pHEA$_{47}$- or BG-pHEA$_{45}$@Au$_4$ solution (500 µL) was concentrated by centrifugation in Amicon Ultra-0.5 centrifugal filter units with an Ultracel-30 membrane. SNAP-AFP was conjugated to the BG-pHEA$_{45}$@Au$_4$; Cys-

AFP was conjugated to Mal-pHEA$_{47}$@Au$_4$; and His-AFP was conjugated to Ni-NTA-pHEA$_{17}$@Au$_4$. To this concentrate was added the same volume of protein solution as was originally concentrated to retain the same concentration of AuNPs. This was left, agitating, for 120 minutes at room temperature. The solutions were concentrated by centrifugation in Amicon Ultra-0.5 centrifugal filter units with an Ultracel-30 membrane, before being re-dispersed in the same volume of pH 7.5 PBS buffer. The nanoparticles were washed in this manner a further 2 times before being re-dispersed in the same final volume of PBS and stored in the fridge until required. Nanoparticle size and dispersity was measured by TEM, UV-Vis spectroscopy and size and zeta potential measurements by DLS.

Recombinant expression of AFPIII

A pET20b-AFPIII plasmid encoding for a hexahistidine-tagged AFPIII from ocean pout (*rQAE isoform*, M1.1HISPET20b) was kindly provided by Peter Davies (Queens University, Kingston, Canada). The plasmid was transformed into competent Escherichia coli BL21(DE3) cells (New England Biolabs). A colony was selected to inoculate 50 mL of LB-medium containing 100 μg/mL ampicillin and was grown overnight at 37 °C under continuous shaking of 180 rpm. The following day, 5 mL of the preculture was added to 500 mL of LB-medium in a 2 L Erlenmeyer flask and grown at 37 °C with a shaking speed of 180 rpm till an OD600 of 0.6 was reached. The temperature was then reduced to 16 °C and IPTG was added to a final concentration of 0.4 mM. The overexpression of the protein was allowed to take place overnight following which the cells were centrifuged at 4000 g for 30 minutes at 4 °C.

Pelleted cells were resuspended in PBS supplemented with Pierce protease inhibitor mini-tablets. The suspension was passed through a STANSTED 'Pressure Cell' FPG12800 homogeniser in order to lyse the cells. The cell lysate was centrifuged at 14,000 g and the supernatant was passed through a 0.45 nm filter and applied to an

IMAC Sepharose 6 Fast Flow (GE Healthcare) column charged with Ni(II) ions and pre-equilibrated with PBS. The column was washed with 20 column volumes of 20 mM imidazole in PBS. Bound AFPIII was eluted using 300 mM Imidazole in PBS. The AFPIII was further purified using a HiLoad 16/600 Superdex 75 pg gel filtration column (GE Healthcare) with PBS as the running buffer. Fractions exclusively containing the AFPIII were pooled and concentrated to 5 mg/mL. Purity was estimated using SDS-PAGE and protein concentration determined using Thermo Scientific Pierce BCA assay kit. This was verified by measuring absorbance at 280 nm and obtaining protein's extinction coefficient [as predicted by ProtParam (http://web.expasy.org/protparam/)] for use in Beer-Lambert law.

Recombinant expression of AFPIII-Cys

The terminal Ala in the pET20b-AFPIII plasmid was mutated to Cys by site-directed mutagenesis using the primer sequence 5'-GTTAAAGGTTACGCTTGTCTCGAGCACCAC-3' and employing Phusion® High-Fidelity DNA Polymerase (New England Biolabs) according to the manufacturer's protocol. The expression and purification was performed as detailed for AFPIII with the exception that 2 mM β-mercaptoethanol was added to all buffers when performing IMAC and gel filtration to prevent the formation of disulfide bonds. The β-mercaptoethanol was removed prior to use by buffer exchanging using PD10 desalting columns (GE Healthcare).

Recombinant expression of AFPIII-SNAP

A genetic fragment encoding for AFPIII fused to a hexahistidine tag was amplified from the pET20b-AFPIII plasmid using 5'-GTACGGATCCAACCAGGCTAGCGTTGTG-3' (BamHI site underlined) as the forward primer and 5'-ATTAGCGGCCGCAGCCGGATCTCAGTG-3' (NotI site underlined) as the reverse primer. The BamHI/NotI digested products were ligated into

a pSNAP-tag® (T7)-2 vector (New England Biolabs). The plasmid was then transformed into competent Escherichia coli BL21(DE3) cells (New England Biolabs). The subsequent expression and purification was performed as detailed for AFPIII.

Ice Recrystallisation Inhibitory activity "splat" assay

The sample was prepared in PBS solution, and a 10 μL was dropped onto a glass microscope coverslip atop an aluminium plate cooled to -78 with dry ice. The thin wafer of ice that forms upon contact with the coverslip was transferred to a cryostage held at -8 ºC using liquid nitrogen, and left to anneal for 30 minutes at this temperature. Three images were taken at different locations on this wafer at 20 x zoom under cross polarizers. Using ImageJ, the number of crystals in the images were counted and the average crystal size per wafer was calculated as mean grain area (MGS) as well as the mean grain length size (MLGS).

Modified Sucrose Sandwich Ice Shaping Assay

Samples dissolved in PBS buffer containing 45% sucrose were sandwiched between two glass coverslips and sealed with immersion oil. Samples were cooled to −50 °C on a Linkam Biological Cryostage BCS196 with T95-Linkpad system controller equipped with a LNP95-Liquid nitrogen cooling pump, using liquid nitrogen as the coolant (Linkam Scientific Instruments UK, Surrey, U.K.). The temperature was then increased to −8 °C and held for 1 hour to anneal. The samples were then heated at 0.5 $°C.min^{-1}$ until few ice crystals remained and then cooled at 0.05 $°C.min^{-1}$ and the shape of ice crystals observed. Micrographs were obtained every 0.1 °C using an Olympus CX41 microscope equipped with a UIS-2 20x/0.45/∞/0−2/FN22 lens (Olympus Ltd., Southend on sea, U.K.) and a Canon EOS 500D SLR digital. Image processing was conducted using ImageJ.

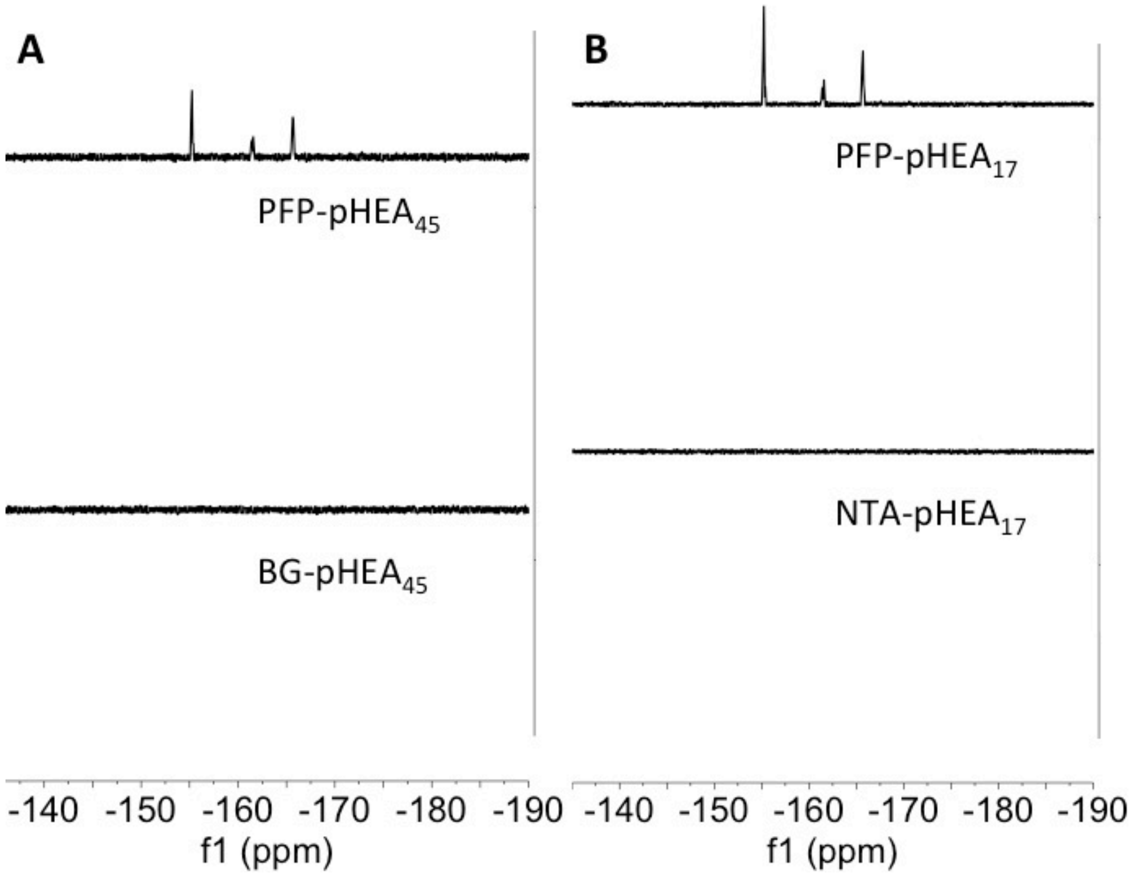

Figure S1: pHEA polymer before and after functionalisation with primary amines: A) PFP-pHEA45 modified to BG-pHEA45, B) PFP-pHEA6 modified to NTA-pHEA6.

In each case the depletion of the three fluorine peaks is observed as evidence of the successful removal of the PFP-group.

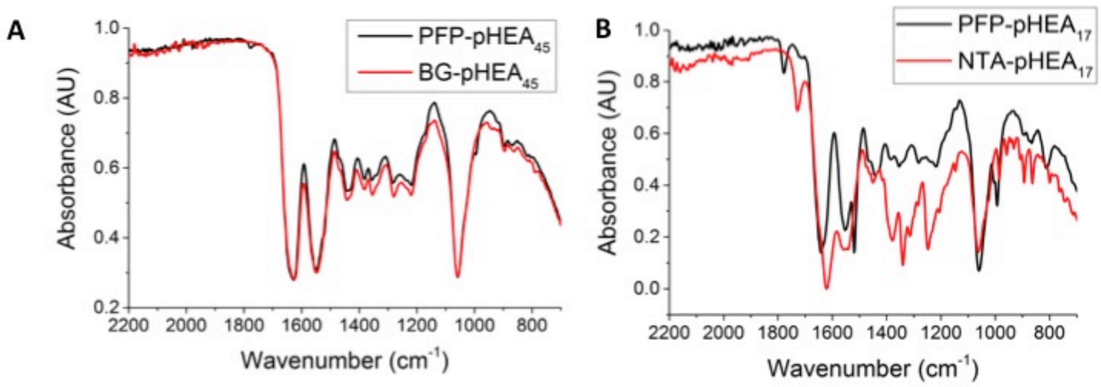

Figure S2: PFP-pHEA polymer before and after functionalisation with primary amines: A) PFP-DMP-terminal pHEA and reaction with BG-amine, B) PFP-DMP-terminal pHEA and reaction with NTA-amine.

In each case the depletion of C-O peak at ~1000 cm^{-1} is observed as evidence of the successful removal of the PFP-group.

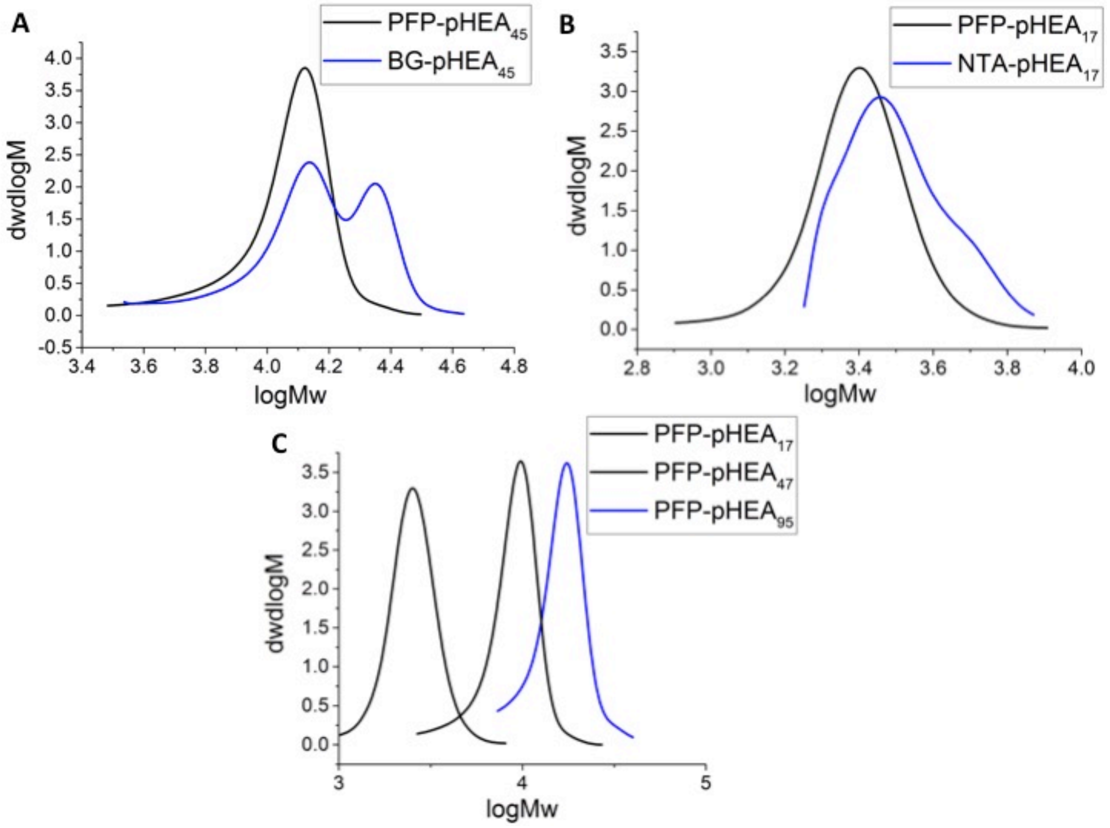

Figure S3: pHEA polymer before and after functionalisation with primary amines: A) PFP-DMP-terminal pHEA and reaction with BG-amine, B) PFP-DMP-terminal pHEA and reaction with BG-amine, C) PFP-pHEA of different chain lengths.

It should be noted that the elution behaviour of the PFP-pHEA polymers changed drastically after end-group modification – the hydrophobic end-group led to tailing in the SEC, and hence the non-symmetric trace was obtained.

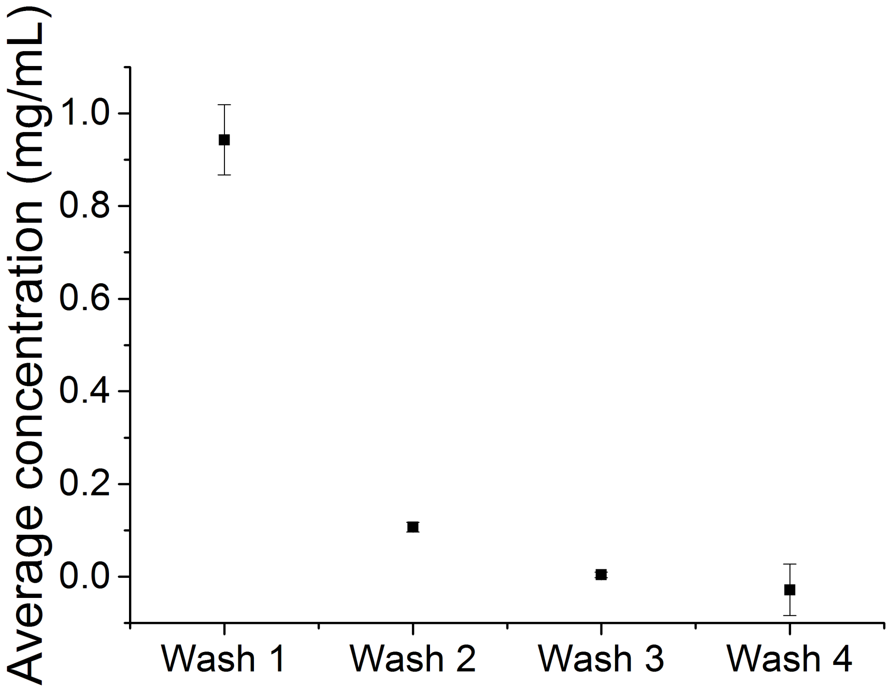

Figure S4: Nanodrop measurements showing depletion of AFP in washes. Protein concentration is on the y-axis. Error bars are standard deviation from n = 3.

Figure S4 shows protein residual protein concentration in the supernatant following washing of the AuNPs, determined using a Nanodrop. There was no detectable protein remaining after 4 washes, confirming the washing method success.

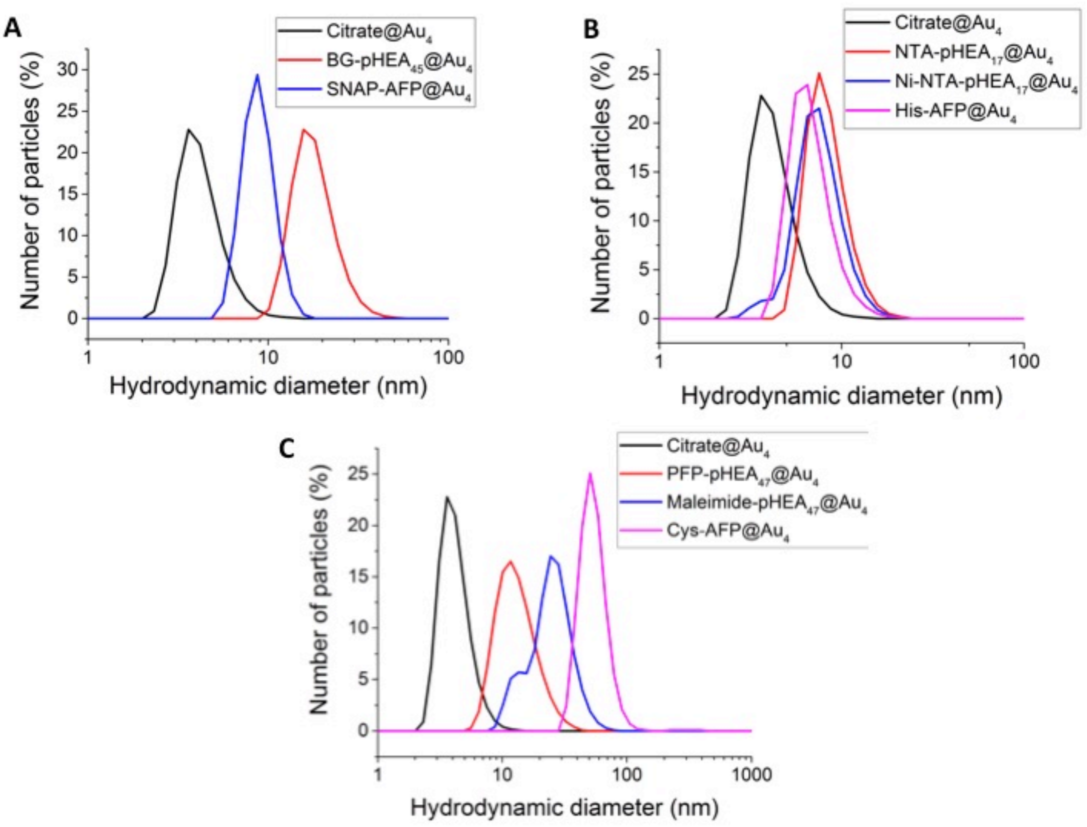

Figure S5: AuNP samples before and after conjugation with polymer and subsequent post-conjugation modification: A) Conjugation of BG-pHEA$_{45}$ followed by SNAP-AFP, B) Conjugation of NTA-pHEA$_{17}$ followed by reaction with nickel chloride (to form Ni-NTA complex), followed by His-AFP, C) Conjugation of PFP-pHEA$_{47}$ followed by reaction to form Maleimide-pHEA$_{47}$, followed by Cys-AFP.

For the SNAP- and His-AFP samples, in each case an increase in size is observed. For the Cys-AFP sample, the size of the maleimide- and Cys-AFP@Au$_4$ showed significant increases in size that resulted in aggregation and sedimentation of the nanoparticles.

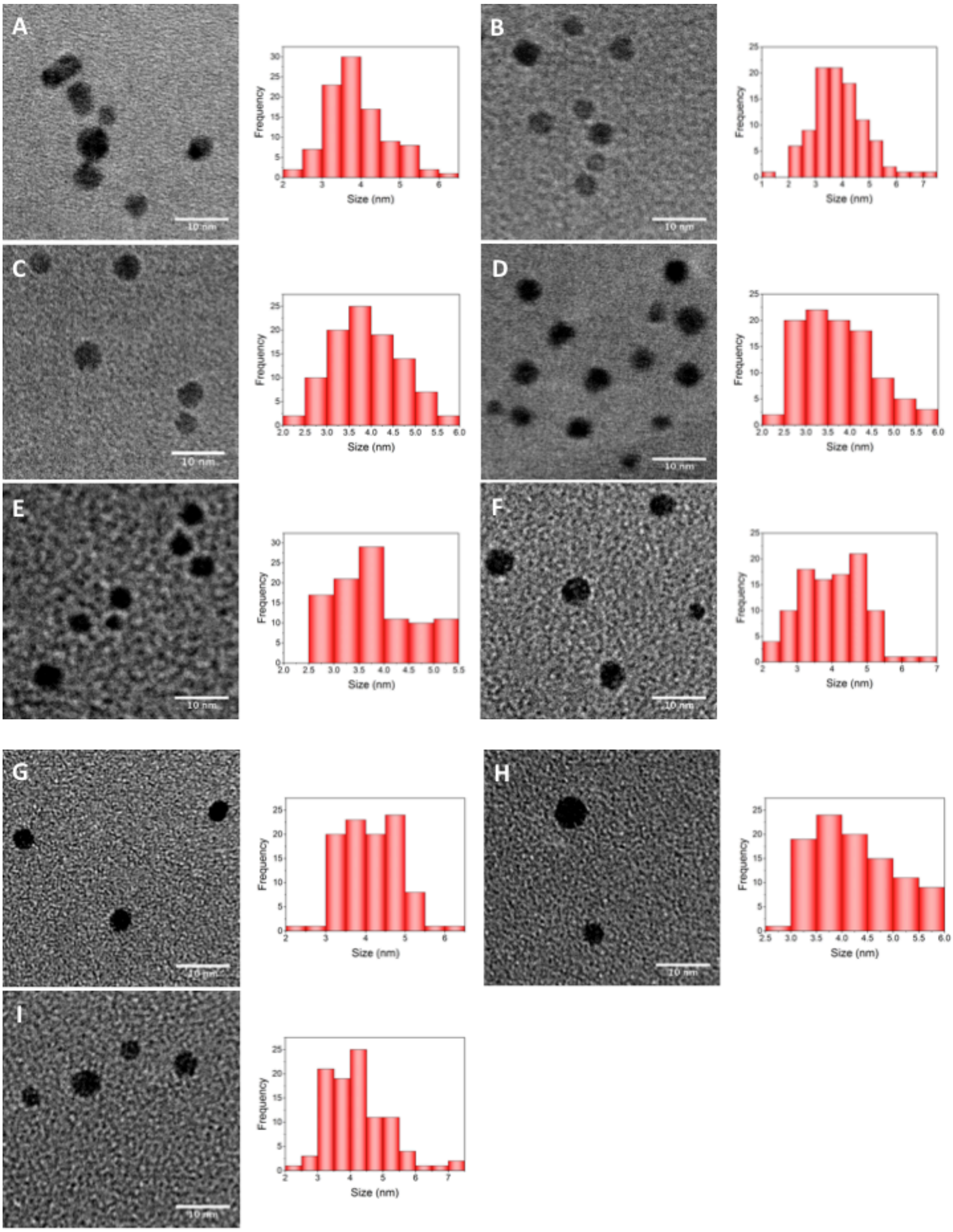

Figure S6: TEM micrographs and size distributions with different coatings: A) Citrate@Au_4, B) BG-$pHEA_{45}$@Au_4, C) SNAP-AFP-BG-$pHEA_{45}$@Au_4, D) NTA-$pHEA_{17}$@Au_4, E) Ni-NTA-$pHEA_{17}$@Au_4, F) His-AFP-Ni-NTA-$pHEA_{17}$@Au_4, G) PFP-$pHEA_{47}$@Au_4, H) Maleimide-$pHEA_{47}$@Au_4, I) Cys-AFP-Maleimide-

pHEA$_{47}$@Au$_4$. Size distributions were calculated with the use of ImageJ to determine the diameters of 100 particles in each sample.

In each sample no significant change in size or uniformity was observed by TEM.

Table S1: Zeta potential measurements for all AuNP samples.

Particle	ζ Potential (mV) [a]	Conductivity (mS/cm) [a]
**Citrate@Au_4**	-19.4 +/- 2.65	0.24 +/- 0.003
**NTA-$pHEA_{17}$@Au_4**	-15.9 +/- 4.77	0.042 +/- 0.0013
**Ni-NTA-$pHEA_{17}$@Au_4**	-6.16 +/- 1.11	11.8 +/- 0.53
**His-AFP-Ni-NTA-$pHEA_{17}$@Au_4**	-7.03 +/- 1.31	18.5 +/- 1.71
**BG-$pHEA_{45}$@Au_4**	-10.2 +/- 1.34	0.052 +/- 0.0021
**SNAP-AFP-BG-$pHEA_{45}$@Au_4**	-4.35 +/- 0.94	18.8 +/- 2.1

[a] Determined as average of 3 readings.

A shift in the mean zeta potential and conductivity confirmed successive surface modification on the AuNP samples. This was most pronounced in the steps involving the initial conjugation of polymer, the reaction with nickel, and upon adding protein to the maleimide-/BG-coated gold.

Table S2: X-ray photoelectron spectroscopy measurements for all AuNP samples.

Particle	C	O	N	Au	Ni	S	F
**Citrate@Au$_4$**	46.41	52.82	0.00	0.33	0.00	0.43	n/a
**NTA-pHEA$_{17}$@Au$_4$**	66.06	23.10	9.21	0.12	0.00	1.51	n/a
**Ni-NTA-pHEA$_{17}$@Au$_4$**	67.40	19.45	9.62	0.06	0.15	3.30	n/a
**His-AFP-Ni-NTA-pHEA$_{17}$@Au$_4$**	63.93	21.98	12.42	0.19	0.12	1.36	n/a
**BG-pHEA$_{45}$@Au$_4$**	68.40	23.65	7.37	0.23	0.00	0.35	n/a
**SNAP-AFP-BG-pHEA$_{45}$@Au$_4$**	58.48	24.74	12.27	0.17	n/a	0.22	n/a

XPS analysis confirmed the conjugation of polymer as an increase in organic character, and the organic character of the sample changed upon the conjugation of antifreeze protein as an increase in nitrogen presence, which suggests that the protein has conjugated to the sample

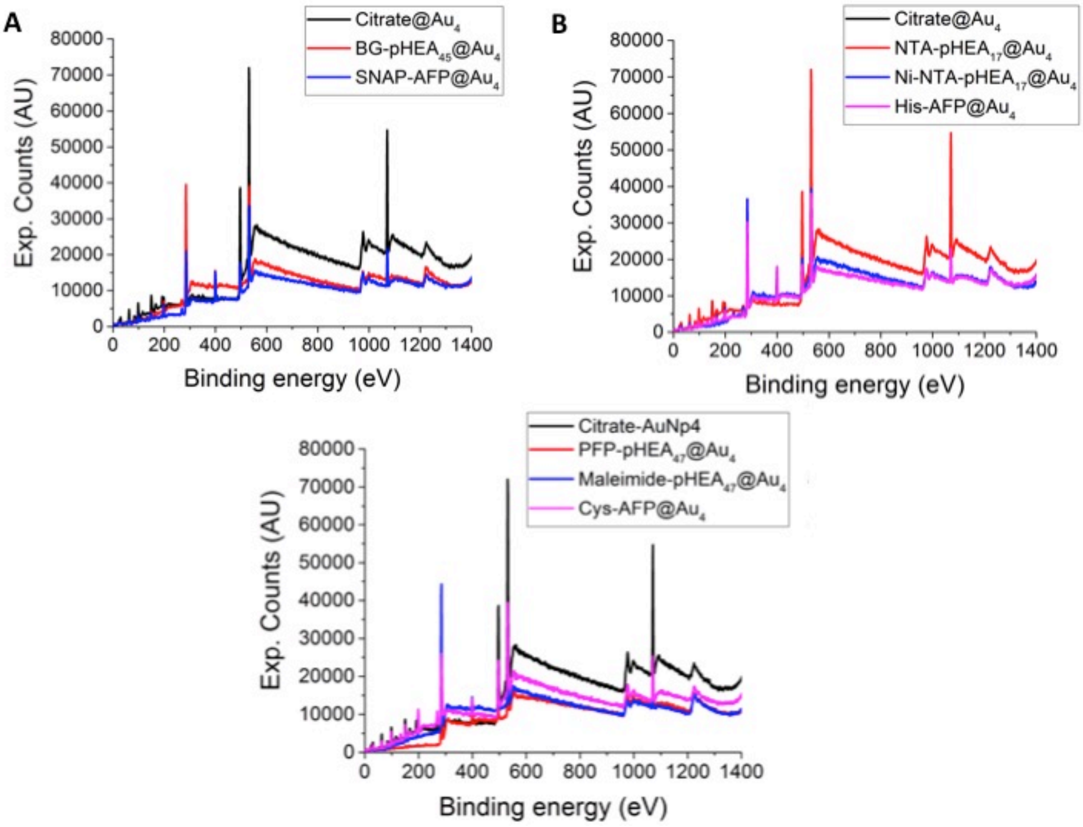

Figure S7: XPS survey scans overlaid for each sequence of samples: A) Citrate@Au_4 to SNAP-AFP-BG-$pHEA_{45}$@Au_4, B) Citrate@Au_4 to His-AFP-Ni-NTA-$pHEA_{17}$@Au_4, C) Citrate@Au_4 to Cys-AFP-Maleimide-$pHEA_{47}$@Au_4.

These raw data survey scans showed a difference in the x-ray photoelectron spectra as coatings were applied to the gold nanoparticles. Although the survey plots for citrate and NTA-$pHEA_{17}$-coated AuNPs were superficially the same in these survey scans, a difference in several peaks was found upon scanning for specific elements such as sulfur and gold.

Table S3: Thermogravimetric Analysis (TGA)

Sample	Coating	Mass (mg) present in 1 mL AuNP
**Citrate@Au_4**	Citrate	0.014
**NTA-$pHEA_{17}$@Au_4**	NTA-$pHEA_{17}$	0.048
**BG-$pHEA_{45}$@Au_4**	BG-$pHEA_{45}$	0.042

The above values were calculated taking into account buffers present (such as PBS present in the protein samples) and against the control of citrate coated particles.

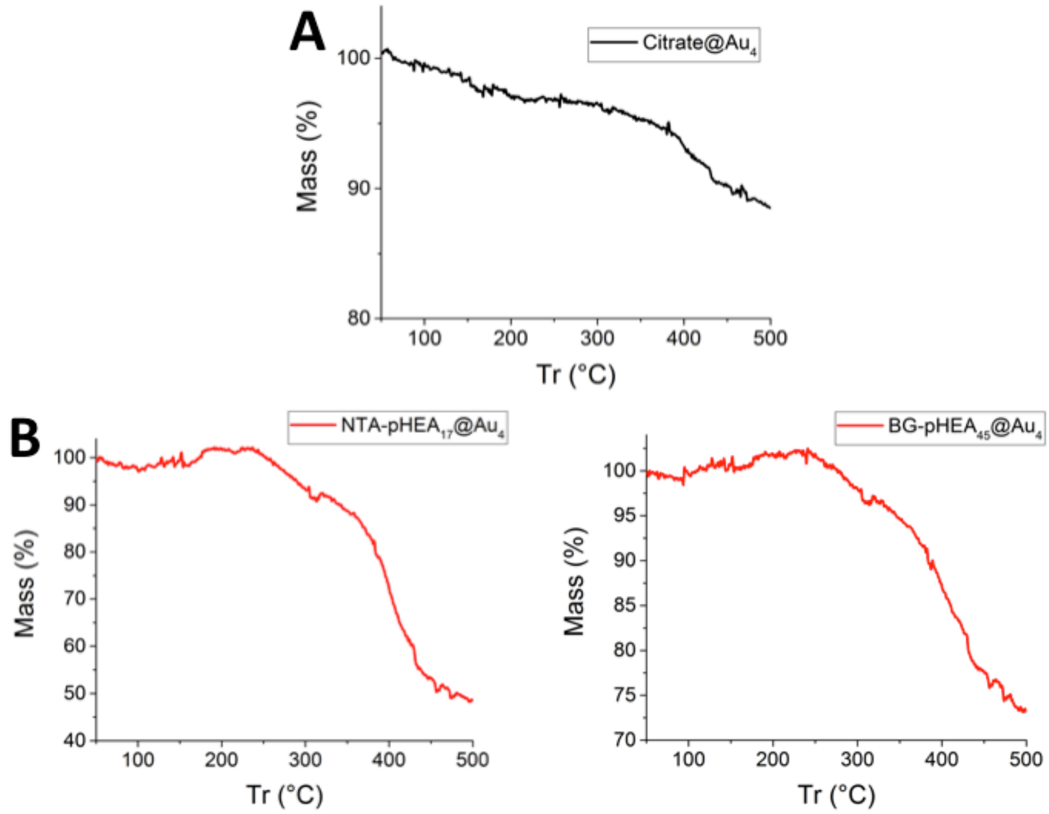

Figure S8: Thermogravimetric analysis curves showing loss of % of mass at increasing temperature for each sample: A) Citrate@Au_4, B) NTA-$pHEA_{17}$@Au_4, C) BG-$pHEA_{45}$@Au_4.

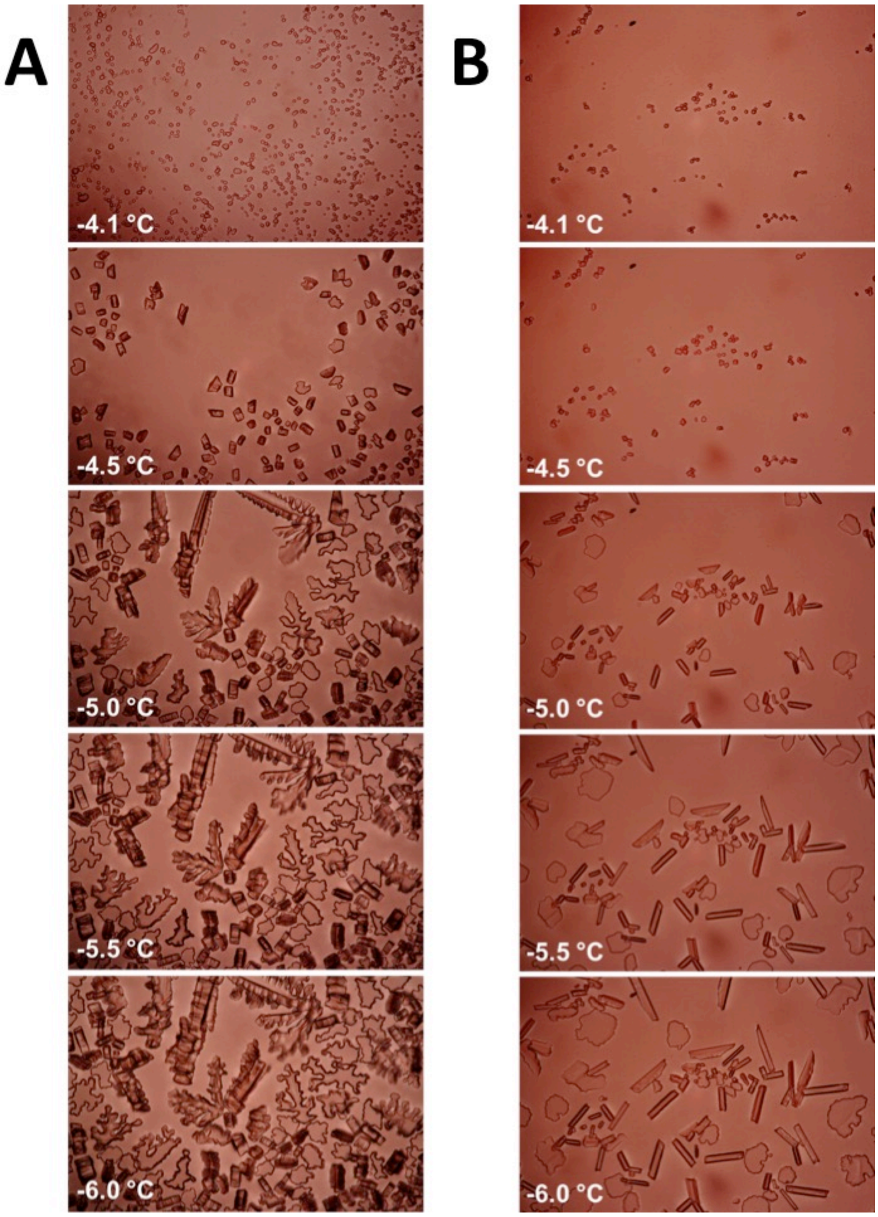

Figure S9: Modified sucrose sandwich ice shaping assay images for the following samples prepared in PBS: A) SNAP-AFP-BG-$pHEA_{45}@Au_4$ at 0.13 $mg.mL^{-1}$ SNAP-AFP concentration, B) SNAP-AFP at 0.10 $mg.mL^{-1}$.

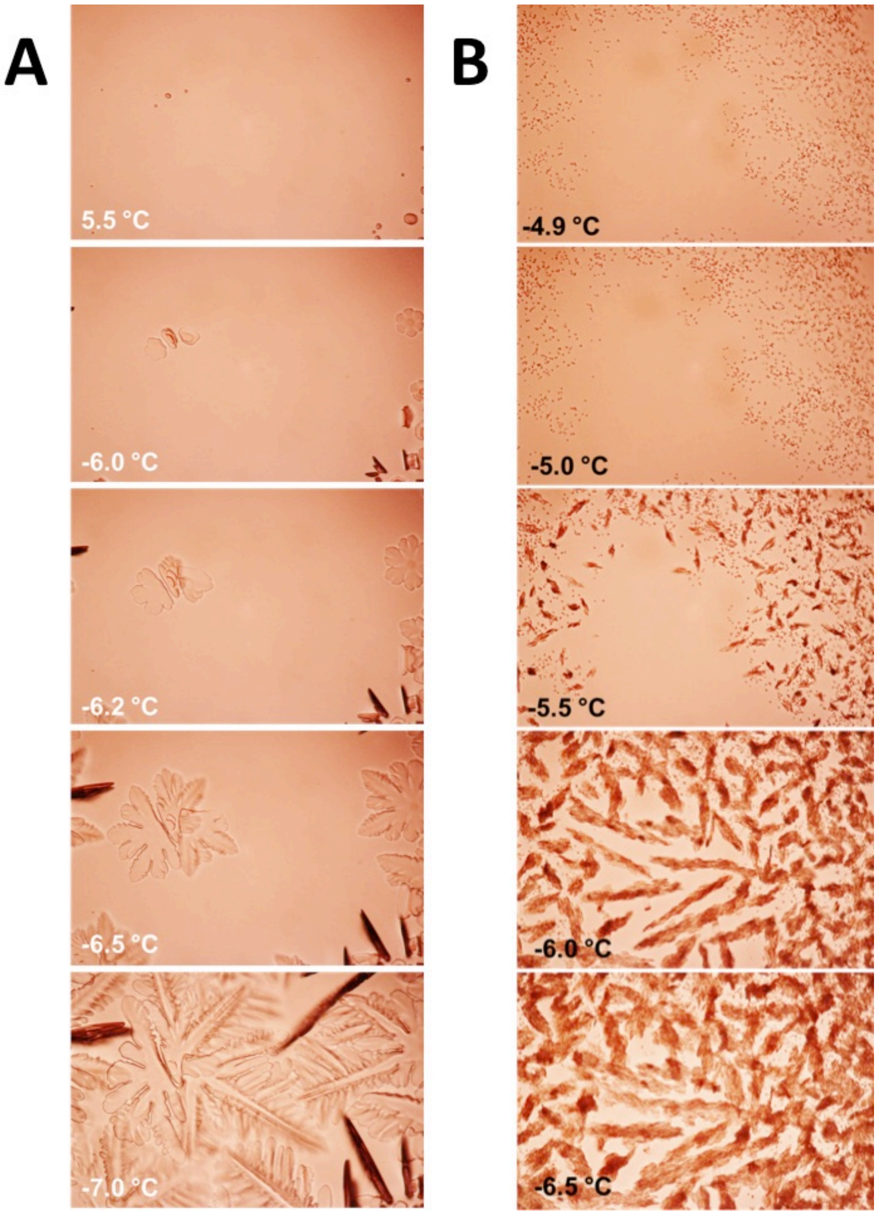

Figure S10: Modified sucrose sandwich ice shaping assay images for the following samples prepared in PBS: A) His-AFP-Ni-NTA-$pHEA_{17}@Au_4$ at 2.3 $mg.mL^{-1}$ His-AFP concentration, B) His-AFP at 2.8 $mg.mL^{-1}$.

These assays show the shape and directionality of ice growth as temperature is gradually decreased. Both recombinant AFP conjugated to the AuNP scaffold showed a branching, feather-like ice growth. The different monomeric recombinant AFPs showed a difference in shape: SNAP-AFP showing straight shard-like shapes and His-AFP showing more curved structure.
